# Investigation of the relationship between suicide probability in inpatients and their psychological symptoms and coping strategies

**DOI:** 10.17712/nsj.2016.4.20150727

**Published:** 2016-10

**Authors:** Dilek Avci, Selma Sabanciogullari, Feride T. Yilmaz

**Affiliations:** *From the Department of Nursing (Avci), Faculty of Health Sciences, Bandirma Onyedi Eylül University, Balikesir, and from the Department of Psychiatric Nursing (Sabanciogullari), Department of Internal Diseases Nursing (Yilmaz), School of Susehri Health High, Cumhuriyet University, Sivas, Turkey*

## Abstract

**Objective:**

To investigate the relationship between suicide probability and psychological symptoms and coping strategies in hospitalized patients with physical illness.

**Methods:**

This cross-sectional study was conducted from April to June 2014 in Bandirma State Hospital, Balikesir, Turkey. The sample of the study consisted of 470 inpatients who met the inclusion criteria and agreed to participate in the study. The data were collected with the Personal Information Form, Suicide Probability Scale, Brief Symptom Inventory and Ways of Coping with Stress Inventory.

**Results:**

In the study, 74.7% were at moderate risk for suicide, whereas 20.4% were at high risk for suicide. According to the stepwise multiple linear regression analysis, sub-dimensions of the Ways of Coping with Stress Inventory and Brief Symptom Inventory were the significant predictors of suicide probability.

**Conclusions:**

The majority of the patients with physical illness were at risk for suicide probability. Individuals who had psychological symptoms and used maladaptive coping ways obtained significantly higher suicide probability scores.

Physical illnesses perceived as a state of crisis by individuals lead to imbalances in one’s life and to the disruption of daily and future plans, creating the need for seeking a new way of adaptation.^1^,^2^ Pain, disability, restrictions in social activities, and financial losses and various factors affecting physical, psychological and social wellbeing are comorbid with physical illnesses.^2^,^3^ This condition leads to various effects on the person such as fear of being dependent on others, fear of losing independence completely, separation anxiety, concern for the future, fear of death, fear of the possibility that the body, or organs or parts of the body would be injured, regret, guilt. Although vary from one illness to another or from one person to another, these negative feelings trigger psychological responses such as anxiety, depression, regression, anger and denial.^4^ In the literature, it has been reported that 41% of people with physical illness are at the risk of developing various mental disorders compared to those who are physically healthy.^1^-^3^ Psychological responses to physical illnesses have direct effects on a person’s ways and capability of coping with the illness.^5^,^6^ When a person feels that the illness poses a threat to his/her bodily integrity and purpose in life, and suffers from the stress due to hospitalization, he/she has difficulty using his/her coping skills. Effects of loss of health or threats to health vary from individual to individual, cause high levels of stress make it difficult to cope. An individual with insufficient resources for successful coping exhibits worsened psychiatric symptoms such as anxiety and depression. This condition affects the person’s adaptation, care, quality of life, duration of the treatment and the prognosis of the disease.^6^-^8^ Physical illness perceived as a state of crisis, and physical, mental and social changes occurring due to the physical illness and the inability to cope with adverse conditions cause suicidal ideation in people.^5^,^9^,^10^

Suicide has been reported to be related with physical illnesses occurring in the early years of life, loss of functional abilities and related job loss, being dependent on someone else, loss of privacy, and distortions in body image.^3^,^10^,^11^ Presence of chronic pain is also known to be an important risk factor for suicide.^12^ Furthermore, comorbid psychiatric history in hospitalized patients with physical illness, and socioeconomic factors increase suicide risk.^2^ In many studies, it has been demonstrated that physical illness significantly increases the risk of suicide.^1^,^3^,^10^,^11^ In the relevant literature, the prevalence of physical illnesses among suicide cases range from 25% to 70%.^4^ A population study conducted in Denmark revealed that 63.5% of the individuals who committed suicide in the last 25 years had physical illness and that the prevalence of suicide risk was 24.4% in hospitalized patients with physical illness.^2^ Similarly, in Turkey, physical illnesses rank first among the reasons of committing suicide and according to the statistics 2011, the rate of individuals who committed suicide due to illness was 19.4%.^13^

Suicide behavior is defined as ending one’s own life willingly and has economic, cultural, social, and legal implications as well as psychiatric, forensic, and public health related results.^14^ Due to the increased suicide rates within the recent years, suicide has become an important public health issue in most countries.^15^,^16^ However, suicide is preventable. Of the health professionals responsible for providing healthcare, nurses play an active and important role in the prevention of suicidal behaviors caused by physical illness.^17^ In the recognition of a suicidal behavior and prevention of suicide in advance, nurses who are always together with inpatients during their treatment in the hospital are supposed to observe how patients perceive the physical illness and how they emotionally react to the illness.

This is also important in terms of patient safety and professional responsibility.^14^,^17^,^18^ Lynch et al^17^ stated that nurses should assess suicide probability by monitoring patients’ physical and psychological conditions during hospitalization in order to prevent suicidal behavior. Similarly, Kellogg et al^14^ pointed out that attempts made by nurses to detect high risk groups are the most important strategy to prevent suicides and they also emphasized that nurses play a key role in the prevention of suicides. In the relevant literature, suicidal ideation^2^,^3^,^10^,^15^ and the reasons for completed suicides among people with physical illness have been investigated.^4^,^19^ A small number of studies examined the relationship between suicide probability and psychiatric symptoms and coping behavior in people with physical illness.^1^,^8^,^18^ Therefore, this present study aimed to determine the psychological symptoms, coping strategies and suicide probability in people with physical illness and to investigate the relationship between these variables, which would contribute to precautions to be taken to prevent suicides, to the development of protective interventions and to studies on this topic.

## Methods

### Study design

This cross-sectional study was conducted between April 2014 and June 2014 in Bandirma State Hospital, Balikesir, Turkey.

### Sample

The population of the present study comprised 585 patients diagnosed with physical illness and hospitalized between April 2014 and June 2014 in a state hospital. In the study, no sample size was calculated. The sample of the study consisted of 470 patients who met the inclusion criteria and agreed to participate in the study. Twenty-eight patients who refused to participate were excluded from the study. Criteria for inclusion were as follows: Agreeing to participate in the study, being older than 18 years, being at least literate, being diagnosed with physical illness, being hospitalized in the internal disease clinics or surgery clinics. Criteria for exclusion were as follows: perception disorders, being hospitalized at the psychiatry, pediatrics, or intensive care services due to illness features and age.

### Data collection instruments

The study data were collected with the Personal Information Form, Suicide Probability Scale, Brief Symptom Inventory (BSI) and Ways of Coping with Stress Inventory (WCSI).

### Personal information form

The form prepared by the researchers through the literature review consists of 19 items questioning some of the socio-demographic characteristics (age, gender, education, marital status, occupation, economic status, residence), psychosocial characteristics (alcohol use, immigration status, previous psychiatric diagnosis, family history of mental disorders, history of suicide attempts, history of suicide in the family), the clinic where the person received treatment, and medical diagnosis of the participants.

### Suicide probability scale (SPS)

This self-report, 36-item, and 4-point Likert-type scale was developed by Cull and Gill in order to assess suicide risk in adolescents and adults. The Turkish validity and reliability of the scale was conducted by Atli et al^16^ in 2009. The scale includes 4 dimensions: hopelessness, suicidal ideation, negative self-evaluation, and hostility. Each of the sub dimensions receives a total score and the sum of all scores gives the overall suicide probability score. Total scores that can be obtained from the scale range from 36 to 144. Higher scores indicate that suicide probability is higher. In addition, total scores are divided into 4 categories in order to assess suicide risk: 0-24 points correspond to the normal group, 25-49 to the mild risk group, 50-74 to the medium risk group, and 75-100 to the high risk group.^16^ In this study, Cronbach’s alpha coefficient of the scale was calculated as 0.85.

### Brief symptom inventory

The inventory was developed by Derogatis in 1992 to assess psychiatric symptoms. The validity and reliability study of the Turkish version of the scale was conducted by Sahin and Durak^20^ in 1994. The 53-item Likert-type scale is scored between 0 and 4 and consists of five subscales: anxiety, depression, negative self, somatization and hostility. High scores obtained from each subscale indicate that individuals experience psychological symptoms frequently. Total scores range from 0 to 212.^20^ In this study, Cronbach’s alpha coefficient of the scale was calculated as 0.96.

### Ways of coping with stress inventory

The inventory was developed by Lazarus and Folkman in 1980. The validity and reliability study of the Turkish version of the scale was conducted by Sahin and Durak^21^ in 1995. The 30-item Likert-type scale is scored between 0 and 3 and consists of five subscales: self-confident approach, optimistic approach, helpless approach, submissive approach, and social support seeking approach. The self-confident, optimistic and social support seeking approaches are considered as effective ways of coping with problems; helpless approach and submissive approach are considered as ineffective/emotion focused ways of coping.^21^ The Cronbach a reliability of the scale in this study was calculated as 0.74.

### Application

The researchers informed the participants about the purpose and importance of the study. The Personal Information Form, SPS, BSI, and WCSI were filled in through one-to-one interviews. The implementation of the data collection tools took almost 30 minutes.

### Ethical approval

The study protocol was designed in compliance with the Declaration of Helsinki. Prior to data collection, necessary approvals and permissions were obtained from the Cumhuriyet University Ethics Committee (Decision number: 04/11) and General Secretariat of the Public Hospitals Union, respectively. The aim of the study was explained to all the study participants and oral consent of the participants was taken.

### Statistical analysis

Statistical analyses were performed using the Statistical Package for Social Sciences Version 16.0 (SPSS Inc., Chicago, IL, USA). Values were expressed as mean±SD or as percentages. Correlations between psychological symptoms, coping characteristics, and suicide probability were computed through the Pearson’s correlation analysis. Correlation between continuous variables were categorized as low (correlation coefficient was between 0.10–0.29), moderate (between 0.30-0.49) and high (>0.50) according to their correlation coefficient values. To identify the independent variables (psychological symptoms, coping strategies) that contribute to suicide probability, stepwise multiple linear regression analysis was performed. The limit of statistical significance was set at *p*-values <0.05.

## Results

The mean age of the participants was 47.14±17.58. Of the participants, 52.8% were female, 64.9% were married, 41.5% graduated from elementary school, and 90.4% lived with their families. Whereas, 48.9% of the participants were receiving treatment at the surgery clinics, 51.1% were receiving treatment at the internal disease clinics. Of the participants, 78.7% had moderate income levels, 67.7% lived in a county, 86% had not consulted a psychiatrist before, 14% were admitted to a psychiatry clinic before, 91.5% had never considered committing suicide, 8.5% had considered it, 96.6% had not attempted to commit suicide and 3.4% had attempted.

The mean suicide probability score of the participants (66.54±11.64) was below average level and the mean score obtained from the negative self subscale (22.77±5.14) was higher than the mean scores obtained from the other subscales. When we categorized the scores obtained from the SPS, we determined that 74.7% of the participants were at moderate risk for suicide and that 20.4% were at high risk. According to the BSI, the psychological condition of the participants was good (40.66±29.32), however, their depression scores (10.66±7.88) were higher than their scores for other psychological symptoms. Regarding the WCSI, the participants mostly used the self-confident approach (13.55±3.82) and the optimistic approach (9.21±2.84) (**Table 1**).

**Table 1:** Distribution of suicide probability scale, brief symptom inventory and ways of coping with stress inventory total and subscale scores.

Variables	mean±SD	Min-Max
*Suicide probability scale*		
Total score	66.54±11.64	37 – 114
Hopelessness	22.12±5.49	12 – 45
Suicidal ideation	10.70±3.55	8 – 31
Negative self-evaluation	22.77±5.14	9 – 34
Hostility	10.93±3.49	7 – 27
*Brief symptom inventory*		
Total score	40.66±29.32	0–212
Anxiety	8.82±7.63	0 – 41
Depression	10.66±7.88	0 – 44
Negative self	7.73±7.23	0 – 46
Somatization	7.08±5.34	0 – 25
Hostility	6.35±4.93	0 – 26
*Ways of coping with stress inventory*		
Self-confident approach	13.55±3.82	0 – 21
Optimistic approach	9.21±2.84	0 – 15
Helpless approach	10.27±4.17	0 – 23
Submissive approach	7.75±2.65	0 – 15
Social support seeking approach	7.11±2.22	0 – 12
*Suicide probability scale groups*	*n (%)*	
0–24 no risk	0	(0.0)
25–49 mild risk	23	(4.9)
50–74 medium risk	351	(74.7)
75–100 high risk	96	(20.4)

In **Table 2**, the relationship between suicide probability scores and WCSI and BSI subscale scores were shown. There was a strong positive correlation (r ranging from 0.40 to 0.66) between the mean total score of suicide probability and BSI subscale scores (*p*<0.001). The mean total score of suicide probability positively correlated with the mean scores for the helpless and submissive coping approaches (r ranging from 0.38 to 0.23) and negatively correlated with the optimistic approach and social support seeking subscale scores (r ranging from -0.13 to -0.15). The hopelessness, suicidal ideation, and hostility subscale scores of the SPS negatively correlated (r ranging from -0.15 to -0.36) with the self-confident approach, optimistic approach, and social support seeking subscale scores of the WCSI; whereas they positively correlated (r ranging from 0.15 to 0.48) with the mean scores for the helpless approach and submissive approach. All subscales of the BSI negatively correlated (r ranging from -0.11 to -0.31) with the self-confident approach, optimistic approach and social support seeking subscales of the WCSI while they positively correlated (r ranging from 0.11 to 0.42) with the submissive and helpless coping approaches.

**Table 2:** Correlations between suicide probability scale, ways of coping with stress inventory and brief symptom inventory.*

Variables	1	2	3	4	5	6	7	8	9	10	11	12	13	14	15
*SPS*															
Total score	-														
Hopelessness	.86**														
Suicidal ideation	.70**	.65**	-												
Negative self-evaluation	.34**	-.01	-.24**	-											
Hostility	.77**	.67**	.64**	-.08	-										
*WCSI*															
Self-confident approach	-.02	-.15**	-.36**	.49**	-.20**	-									
Optimistic approach	-.15**	-.24**	-.35**	.37**	-.32**	.70**	-								
Helpless approach	.38**	.48**	.37**	-.15**	.35**	-.08	-.12**	-							
Submissive approach	.23**	.25**	.20**	.00	.15**	.03	.15**	.47**	-						
Support seeking approach	-.13**	-.21**	-.27**	.26**	-.22**	.23**	.21**	-.19**	-.17**	-					
*BSI*															
Anxiety	.65**	.64**	.66**	-.12**	.66**	-.26**	-.30**	.37**	.20**	-.23**	-				
Depression	.60**	.67**	.62**	-.15**	.54**	-.26**	-.27**	.42**	.24**	-.16**	.83**	-			
Negative self	.66**	.67**	.73**	-.16**	.64**	-.31**	-.31**	.37**	.17**	-.20**	.85**	.79**	-		
Somatization	.40**	.43**	.47**	-.12**	.38**	-.16**	-.12**	.31**	.22**	-.11*	.70**	.71**	.62**	-	
Hostility	.63**	.60**	.55**	-.06	.69**	-.15**	-.24**	.31**	.11*	-.17**	.73**	.68	.70**	-.54**	-

1 - SPS total, 2 - Hopelessness, 3 - Suicidal ideation, 4 - Negative self-evaluation, 5 - Hostility, 6 - Self-confident approach, 7 - Optimistic approach, 8 - Helpless approach, 9 - Submissive approach, 10 - Social support seeking approach, 11 - Anxiety, 12 - Depression, 13 - Negative self, 14 - Somatization, 15 - Hostility,

* *p*<0.05;

** *p*<0.01, SPS - Suicide probability scale, WCSI - Coping with Stress Inventory, BSI - Brief symptom inventory

The stepwise multiple linear regression analysis, which examined suicide probability according to the BSI and the WCSI, is summarized in **Table 3**. The t-test results, which indicate the significance of regression coefficients, revealed that the WCSI and the BSI subscale variables were significant predictors of suicide probability (R=0.74, R^2^=0.55, F=71.45, *p*<0.001). WCSI and the BSI subscale variables, together, explain 55% of the variance in suicide probability. According to the standardized regression coefficient (ß), variables that affect suicide probability are the negative self subscale (ß=0.35) of the BSI, the hostility subscale (ß=0.25), the anxiety subscale (ß=0.24), the self-confident approach subscale (ß=0.22) of the WCSI, the somatization subscale (ß=-0.14) of the BSI, the helpless approach subscale (ß=0.09) of the WCSI, the optimistic approach (ß=-0.08), and the submissive approach (ß=0.08) respectively. Variables that were excluded from the stepwise multiple linear regression analysis were the depression subscale of the BSI and the social support seeking subscale of the WCSI.

**Table 3:** Stepwise regression analysis of suicide probability.*

Variables	B	SE	ß	t	*p*-value
*BSI*					
Anxiety	0.37	0.10	0.24	3.566	<0.001
Negative self	0.57	0.09	0.35	5.763	<0.001
Somatization	-0.32	0.09	-0.14	-3.270	0.001
Hostility	0.60	0.11	0.25	5.417	<0.001
*WCSI*					
Self-confident approach	0.69	0.13	0.22	5.074	<0.001
Optimistic approach	-0.34	0.19	-0.08	-1.808	0.071
Helpless approach	0.25	0.10	0.09	2.356	0.019
Submissive approach	0.36	0.16	0.08	2.221	0.027

* R=0.74,

R^2^=0.55, Durbin-Watson, 1.752 (*p*<0.001), WCSI - Coping with Stress Inventory, BSI - Brief symptom inventory, SE - standard error

## Discussion

The results of the current study were discussed from 2 perspectives: Suicide probability, psychological condition, and coping status. People with physical illness are at high risk for suicide.^2^,^19^ In this study, it was determined that the suicide probability of the participants was at moderate levels and that the majority of the participants were at high risk for suicide. In a study by Marusic and Goodwin^10^ conducted with 415 people with physical illness, 16% of the participants had suicidal ideation. Also, in studies that examine completed or attempted suicides, it was reported that having physical illness is a risk factor for suicide.^4^,^19^,^22^ Previous studies pointed to the suicide rates at general hospitals where people with physical illness receive treatment. In a study conducted in Finland, the rate of suicides committed at 26 general hospitals was 1.9% compared to all suicide cases.^19^ In people with physical illness, reasons including increased length and frequency of hospitalization, relapses, and deterioration of mental health and quality of life can increase suicidal ideation. In this context, it is important to assess individuals who are newly diagnosed for psychological symptoms and suicide risk starting from the onset of illness. In a study conducted in the United Kingdom, approximately half of the elderly patients who committed suicide visited their family doctors prior to their death and more than 5% of these visits were due to physical complaints.^22^ Therefore, nurses who are on duty for 24 hours should monitor patients with physical illness for suicide risk during their hospitalization and should prevent suicide by taking the necessary precautions in order to provide patient safety and to fulfill professional responsibilities.

Short-term or long-term medicine use, organ damage, or comorbid diseases cause physical illnesses which affect one’s mental health.^7^ This condition may also have a negative impact on disease prognosis. In the present study, it was determined that depression was the most frequently observed psychological symptom among people with physical illness. Other studies demonstrated that people with a chronic disease are at high risk for mental disorders.^1^-^3^ Since better psychiatric conditions will have a positive effect on recovering from physical illness as well as preventing the development of physical and psychiatric complications, it is important for nurses to detect and meet psychiatric needs of patients in addition to physical needs.

Being ill is a major source of stress. Individuals who face illness develop various coping approaches in order to cope with the illness and to minimize the negative aspects of the illness.^23^ Each patient’s personality, illness perceptions, coping strategies, and responses to illness vary. In this study, the participants used the self-confident and optimistic coping approaches more frequently. This is a positive finding and can be explained by the fact that most of the participants were past the middle ages and therefore had more experience regarding illness. In addition, most participants lived with their families, which provided a social support network for them. The use of effective coping styles is important in terms of disease management. In a study conducted with hemodialysis patients, the patients were determined to use positive coping styles.^18^ In another study, the participants used support systems for coping more frequently.^6^ The relationships between suicide probability, psychological condition, and coping. The development of psychiatric disorders in people with physical illness facilitates suicide.^19^ In the study, suicide probability increased as psychiatric disorders intensified. In a study by Qin et al,^1^ physical and psychiatric disorders are important risk factors for suicide and suicide rates of people with physical illness and comorbid psychiatric disorders are high. In other studies, levels of suicidal ideation increased as the severity of depression increased.^18^,^24^ Retrospective studies also revealed that most of the suicide cases in general hospitals had psychiatric disorders.^19^,^25^ In a meta-analytic study, the suicide rate was 10 times more common among people with psychiatric disorders compared to the general population.^26^ Hospitalized people show increases in physical symptoms, limitations, and the need for medicine. This condition can have a negative impact on the psychological state of people with physical illness and can increase suicidal ideation. The idea of ending one’s life can be a source of stress for some people and can be a way for relieving stress for others.^27^ Detecting the coping styles of people with physical illness helps determine treatment goals and therapeutic effectiveness and also helps prevent the emergence of additional problems such as suicide.^23^ In the current study, it was determined that people who use helpless and submissive coping styles had increased suicide probability and that those who used an optimistic style and those who sought social support had decreased levels of suicide probability. In a study by Marusic and Goodwin,^10^ the use of coping strategies such as avoidance and emotional procrastination was determined to be associated with suicidal ideation. In another study, active coping, planning, and positive reframing were found to be negatively correlated with suicidal ideation.^18^ Other studies also determined that people who attempted suicide used effective coping styles to a lesser extent^8^,^9^,^27^,^28^ and that they had inadequate problem-solving skills.^29^ According to the study results, improving one’s coping behaviors is an important indicator of suicide prevention. In addition, in the study, those who exhibited hopelessness, suicidal ideation, and hostility adopted helpless and submissive coping styles more frequently. In this context, nurses should evaluate the psychosocial characteristics of individuals during the treatment process.

In this study, psychological symptoms and coping styles were determine to have significant effects on suicide probability. Half of the individuals considered suicide due to the presence of psychological symptoms and ineffective coping styles. In a study by Ozguven et al^30^ people with high levels of anxiety symptoms and with precipitous problem-solving skills were at high risk for suicide attempts. In a study by Doucet and Letourneau,^9^ emotion and avoidance-focused coping styles precipitated suicidal ideation among people with postpartum depression and problem-focused coping strategies did not precipitate suicidal ideation. The study results underline the importance of nurses’ roles in suicide prevention, which includes detecting psychological symptoms and promoting effective coping styles in patients.

### This study has some limitations

One of its limitations is that because the study was conducted with a relatively small group of patients receiving inpatient treatment at one health center and the results obtained from this study are applicable only to the study population and cannot be generalized to all patients. Another limitation of the study is that the study results are limited to the data obtained from the scales based on self-expression.

In conclusion, approximately all individuals with physical illness are at risk for suicide probability. People who exhibit psychological symptoms and use inadequate coping styles have higher suicide probability. Therefore, nurses should monitor patients for psychological symptoms beginning from the onset of physical illness and should promote effective coping styles among these patients. In patient care provided by nurses, spending enough time with patients and talking to them can facilitate the detection of their psychological condition and suicide probability. In addition, providing regular in-service training composed of detection of psychological symptoms, promotion of effective coping styles and detection of suicide probability for health professionals and particularly for nurses can prevent adverse events. It is suggested that, in addition to psychological symptoms and coping styles, other factors that can cause suicide should be investigated in larger populations in order to prevent suicide among people with physical illness.

Implications of Findings for Future Research. Physical disorders can cause physical, mental, social and economic losses by affecting the entire life of an individual. Therefore, the presence of illness is a risk factor for suicide across all age groups. However, in the literature, the number of studies which examine suicide risk in people with physical illness is limited, and studies only investigated affecting factors of completed suicides. In this context, conducting holistic evaluations of people with physical illness, determining risk factors and preventive factors for suicide and conducting research with large samples in the field as well as treatment centers will contribute to taking precautions against suicide.

## References

[1] Qin P, Hawton K, Mortensen PB, Webb R (2014). Combined effects of physical illness and comorbid psychiatric disorder on risk of suicide in a national population study. Br J Psychiatry.

[2] Qin P, Webb R, Kapur N, Sørensen HT (2013). Hospitalization for physical illness and risk of subsequent suicide: a population study. J Intern Med.

[3] Kaplan MS, McFarland BH, Huguet N, Newsom JT (2007). Physical illness, functional limitations, and suicide risk: a population-based study. Am J Orthopsychiatry.

[4] Ekici G, Savas HA, Çıtak S (2001). Two important risk factors ın committed suicides: existance of physical illness and inadequacy of psychiatric treatment. Bull Clin Psychopharmacol.

[5] Kim SM, Han DH, Trksak GH, Lee YS (2014). Gender differences in adolescent coping behaviors and suicidal ideation: findings from a sample of 73,238 adolescents. Anxiety Stress Coping.

[6] Jakobsson Larsson B, Nordin K, Askmark H, Nygren I (2014). Coping strategies among patients with newly diagnosed amyotrophic lateral sclerosis. J Clin Nurs.

[7] Harter MC, Conway KP, Merikangaz KR (2003). Associations between anxiety disorders and physical illness. Eur Arch Psychiatry Clin Neurosci.

[8] Foroughipour M, Mokhber N, Azarpajooh MR, Taghavi M, Modarres GM, Akbarzadeh F (2013). Coping mechanisms, depression and suicidal risk among patients suffering from idiopathic epilepsy. Int J High Risk Behav Addict.

[9] Doucet S, Letourneau N (2009). Coping and suicidal ideations in women with symptoms of postpartum depression. Clinical Medicine Insights: Reproductive Health.

[10] Marusic A, Goodwin RD (2006). Suicidal and deliberate self-harm ideation among patients with physical illness: the role of coping styles. Suicide Life Threat Behav.

[11] Scott KM, Hwang I, Chiu WT, Kessler RC, Sampson NA, Angermeyer M (2010). Chronic physical conditions and their association with first onset of suicidal behavior in the world mental health surveys. Psychosom Med.

[12] Wilson KG, Kowal J, Henderson PR, McWilliams LA, Péloquin K (2013). Chronic pain and the interpersonal theory of suicide. Rehabil Psychol.

[13] Turkey Statistical Institute (TSI) (2011). Suicide Statistics.

[14] Kellogg KJ, Kaur S, Blank WC (2014). Suicide in corrections: an overview. Dis Mon.

[15] Brinkman TM, Zhang N, Recklitis CJ, Kimberg C, Zeltzer LK, Muriel AC (2014). Suicide ideation and associated mortality in adult survivors of childhood cancer. Cancer.

[16] Atli Z, Eskin M, Dereboy C (2009). The validity and the reliliability of Suicide Probability Scale (SPS) in clinical sample. Clinic Psychiatry.

[17] Lynch MA, Howard PB, El-Mallakh P, Matthews JM (2008). Assessment and management of hospitalized suicidal patients. J Psychosoc Nurs Ment Health Serv.

[18] Keskin G, Engin E (2011). The evaluation of depression, suicidal ideation and coping strategies in haemodialysis patients with renal failure. J Clin Nurs.

[19] Suominen K, Isometsä E, Heilä H, Lönnqvist J, Henriksson M (2002). General hospital suicides--a psychological autopsy study in Finland. Gen Hosp Psychiatry.

[20] Sahin NH, Durak A (1994). The validity, reliability and factor structure of the Brief Symptom Inventory. Turkish Journal of Psychiatry.

[21] Sahin NH, Durak A (1995). Ways of Coping with Stress Inventory: adapting to college students. Turkish Journal of Psychology.

[22] Harwood DM, Hawton K, Hope T, Jacoby R (2000). Suicide in older people: mode of death, demographic factors, and medical contact before death. Int J Geriatr Psychiatry.

[23] Agargun MY, Besiroglu L, Kiran UK, Ozer OA, Kara H (2005). The psychometric properties of The COPE Inventory in Turkish sample: a preliminary research. Anatolian Journal of Psychiatry.

[24] Goodwin RD, Kroenke K, Hoven CW, Spitzer RL (2003). Major depression, physical illness, and suicidal ideation in primary care. Psychosom Med.

[25] Dhossche DM, Ulusarac A, Syed W (2001). A retrospective study of general hospital patients who commit suicide shortly after being discharged from the hospital. Arch Intern Med.

[26] Chesney E, Goodwin GM, Fazel S (2014). Risks of all-cause and suicide mortality in mental disorders: a meta-review. World Psychiatry.

[27] Konkan R, Erkus GH, Guclu O, Senormanci O, Aydin E, Ulgen MC (2014). Coping strategies in patients who had suicide attempts. Archives of Neuropsychiatry.

[28] Tel H, Uzun S (2003). Social support and coping with stress status of patients admitted to the emergency room with a suicide attempt. Anatolian Journal of Psychiatry.

[29] Pollock LR, Williams JM (2004). Problem-solving in suicide attempters. Psychol Med.

[30] Ozguven HD, Soykan C, Haran S, Gencoz T (2003). Importance of problem solving skills, perceived social support, and depression and anxiety symptoms on suicide attempts. Turkish Journal of Psychology.

